# Environmental cleaning in operating rooms: A systematic review from the human factors engineering perspective

**DOI:** 10.1017/ash.2023.317

**Published:** 2023-09-29

**Authors:** Anping Xie, Hugo Sax, Oluseyi Daodu, Lamia Alam, Marium Sultan, Clare Rock, Shawna Perry, Ayse Gurses

## Abstract

**Background:** Environmental cleaning is critical in preventing pathogen transmission and potential consecutive healthcare-acquired infections. In operating rooms (ORs), multiple invasive procedures increase the infectious risk for patients, making proper cleaning and disinfection of environmental surfaces of paramount importance. A human-factors engineering (HFE) approach emphasizing the impact of the entire work system on care processes and outcomes has been proposed to improve environmental cleaning. Using the lens of this HFE approach, we conducted a systematic review to synthesize existing evidence and identify gaps in the literature on OR cleaning. **Methods:** The systematic review was guided by the Preferred Reporting Items for Systematic Reviews and Meta-Analyses (PRISMA) guidelines and limited to English-written, peer-reviewed journal articles reporting empirical studies on OR cleaning. Figure 1 shows the flowchart of study search and screening. The following data were extracted from each included article: (1) general information of the article (eg, first author, title, journal, year of publication) and (2) characteristics of the study (eg, country, objectives, design, outcome measures and measuring techniques, findings, funding source). In addition, work-system elements (eg, people, tasks, tools and technologies, physical environment, organizational conditions) and cleaning processes (eg, turnover cleaning, terminal cleaning) addressed in each included studywere coded based on the Systems Engineering Initiative for Patient Safety (SEIPS) model. The methodological quality of included studies using a (non)randomized controlled design was assessed using the version 2 of the Cochrane risk-of-bias tool for randomized trials. **Results:** In total, 35 studies were included in this review, among which 10 examined the effectiveness of OR cleaning in reducing environmental contamination (Fig. 2), 1 examined the compliance of OR cleaning practices (Fig. 3), and 24 examined interventions for improving OR cleaning effectiveness and/or compliance (Fig. 4). Figure 5 summarizes the characteristics of the included studies. **Conclusions:** In this review, OR cleaning was inconsistently performed in practice, and mixed findings were reported regarding the effectiveness of OR cleaning in reducing environmental contamination. No study has systematically examined work-system factors influencing OR cleaning. Efforts to improve OR cleaning focused on cleaning tools and technologies (eg, ultraviolet light) and staff monitoring and training. Interventions targeting the broader work system influencing the cleaning processes are lacking. The scientific rigor of the included studies was modest. Most studies were either commercially funded or did not reveal their funding sources, which might introduce a desirability bias.

**Financial support:** This study was funded by the Centers for Disease Control and Prevention.

**Figure:**
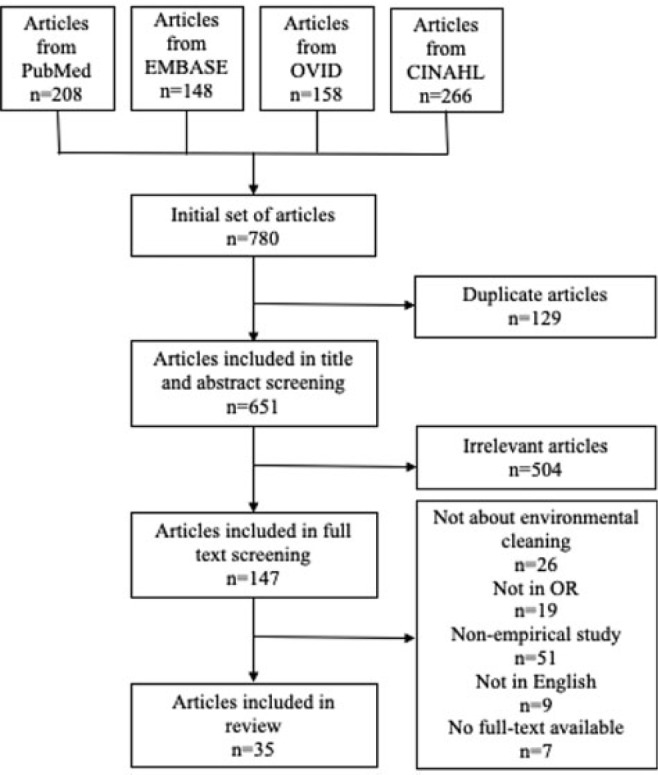

**Figure:**
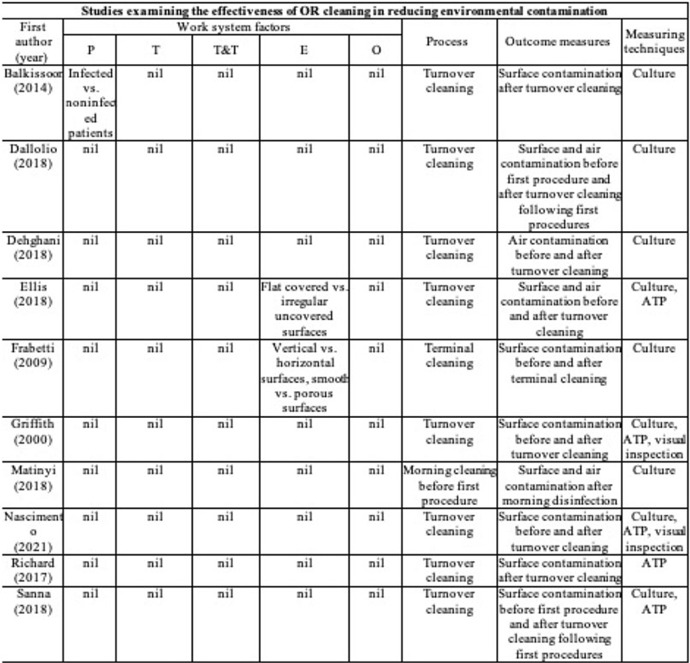

**Figure:**
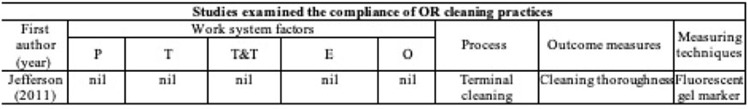

**Figure:**
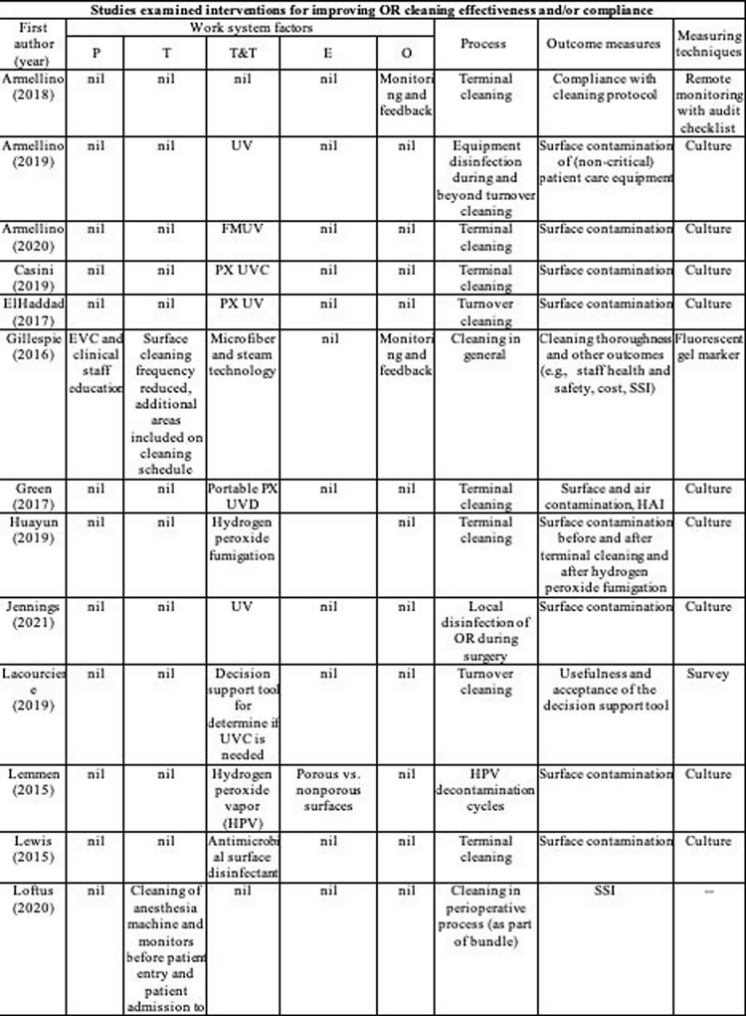

**Figure:**
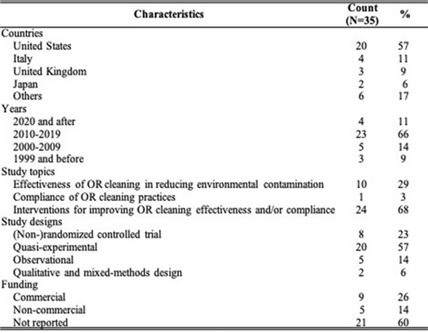

**Disclosures:** None

